# Supplementary material for: The interactome of intact mitochondria by cross-linking mass spectrometry provides evidence for coexisting respiratory supercomplexes
Source: Mol Cell Proteomics. 2017 Dec 8;17(2):216–32. doi: 10.1074/mcp.RA117.000470 (PMC5795388; doi:10.1074/mcp.RA117.000470)
Supplement: Supplemental Data [file supp_RA117.000470_133922_0_supp_23978_fzffwf.zip › spectra_annotation/mito_spectra_annotation/433-2-6-1-14-1.pdf]

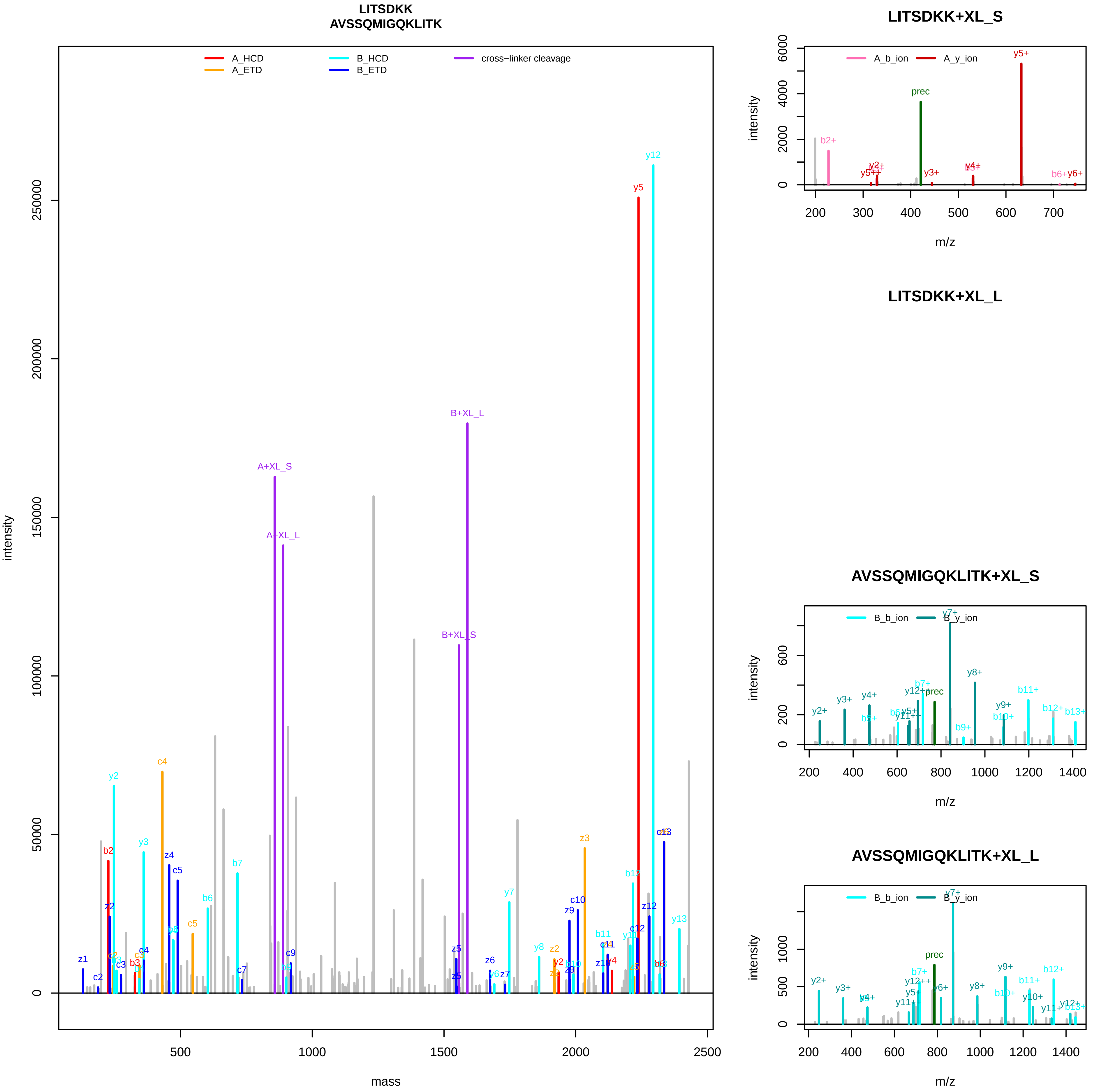

LITSDKK
AVSSQMIGQKLITK
A_HCD
A_ETD
B_HCD
B_ETD
cross−linker cleavage
intensity
mass
0
50000
100000
150000
200000
250000
500
1000
1500
2000
2500
A+XL_S
A+XL_L
B+XL_S
B+XL_L
y12
y5
c4
y2
y3
b2
z2
z4
c5
b7
b6
c5
z3
c10
z9
y7
y8
z12
b12
y13
c13
z1
c2
c3
c4
b3
c7
c9
z5
z6
z7
c11
b11
c12
LITSDKK+XL_S
A_b_ion
A_y_ion
intensity
m/z
0
2000
4000
6000
200
300
400
500
600
700
prec
y5+
b2+
y2+
y3+
y4+
y6+
b6+
LITSDKK+XL_L
AVSSQMIGQKLITK+XL_S
B_b_ion
B_y_ion
intensity
m/z
0
200
600
200
400
600
800
1000
1200
1400
y7+
y8+
prec
b7+
b11+
y9+
y2+
y3+
y4+
b12+
b13+
b9+
AVSSQMIGQKLITK+XL_L
B_b_ion
B_y_ion
intensity
m/z
0
500
1000
200
400
600
800
1000
1200
1400
y7+
prec
b7+
y9+
b12+
b11+
y8+
y6+
y2+
y3+
y4+
